# Pre-existing autoimmune disease as a risk factor for immune-related adverse events in cancer patients receiving immune checkpoint inhibitors

**DOI:** 10.1371/journal.pone.0306995

**Published:** 2024-07-16

**Authors:** Hidetoshi Sumimoto, Satoshi Noda, Hiroyoshi Koide, Yutaro Douke, Kosuke Sakai, Akihito Nishikawa, Azumi Tomioka, Maki Hori, Hiromi Nakato, Yuri Kimura, Aya Tokuda, Atsushi Takano, Koji Teramoto, Satoshi Murata, Yataro Daigo

**Affiliations:** 1 Department of Medical Oncology, Shiga University of Medical Science, Otsu, Shiga, Japan; 2 Cancer Center, Shiga University of Medical Science, Otsu, Shiga, Japan; 3 Center for Advanced Medicine Against Cancer, Shiga University of Medical Science, Otsu, Shiga, Japan; 4 Department of Pharmacy, Shiga University of Medical Science Hospital, Otsu, Shiga, Japan; 5 Nursing Department, Shiga University of Medical Science Hospital, Otsu, Shiga, Japan; 6 Center for Antibody and Vaccine Therapy, Research Hospital, Institute of Medical Science, The University of Tokyo, Tokyo, Japan; Xi’an Jiaotong University Medical College First Affiliated Hospital Department of Medical Oncology, CHINA

## Abstract

Immune checkpoint inhibitors (ICIs) have been widely used as standard therapies for various cancers. However, in 20–30% of cases, ICIs can lead to immune-related adverse events (irAEs), which sometimes require discontinuation of treatment. Due to the increased risk of irAEs, patients with pre-existing autoimmune diseases (AI) are often advised against receiving ICIs. However, there has not been sufficient objective risk assessment for AI. In our study, we conducted logistic regression analysis to assess the risk of irAEs by analyzing 478 cases that received anti-PD-(L)1 Ab and/or anti-CTLA4 Ab at our hospital between April 3, 2017, and May 24, 2022. Among these cases, 28 (5.9%) had pre-existing AI. We selected several independent factors for analysis: gender, age, performance status (PS), cancer type, type of ICI, type of combined anti-cancer agents, best overall response, and pre-existing AI. The adjusted odds ratio (OR) of AI for irAE occurrence was 2.52 [95% CI: 1.08–5.86] (p = 0.033), and the adjusted OR of AI for ICI discontinuation due to irAE was 3.32 [1.41–7.78] (p = 0.006). Patients with pre-existing AI experienced a significantly shorter irAE-free survival time compared to those without AI (median irAE-free survival: 5.7 months [95% CI: 3.5–7.8] vs 10.4 months [95% CI: 7.9–12.9], respectively, p = 0.035). Frequently observed irAEs in full ICI cohort, such as dermatologic issues (7.5%), pneumonitis (7.1%), hepatitis (4.6%), and hypothyroidism (4.2%), were often accompanied by pre-existing AI. Furthermore, pre-existing AI flared up in 6 cases (37.5% in AI-positive irAE-positive cases). The activity of AI was not related to the occurrence of irAEs. Grade 3 or higher irAEs were observed in 6 out of 20 (30.0%) cases in AI-accompanied patients complicated with irAEs. Although having a complicated AI increases the risk of irAEs, it may not necessarily be a contraindication for ICI treatment if closely monitored. (292<300 characters).

## Introduction

The use of immune-checkpoint inhibitors (ICIs) has revolutionized the prognosis of patients with various malignancies, significantly improving their outcomes [1]. ICIs have become standard therapy options at different stages of treatment, either as standalone treatments or in combination with other therapeutic approaches [2]. However, the administration of ICIs can sometimes lead to immune-related adverse events (irAEs), resulting in treatment discontinuation, severe organ damage, or even death [3]. Early detection and effective management of irAEs are crucial in order to minimize these complications. Patients with pre-existing autoimmune diseases (AI) have typically been excluded from clinical trials, presumably due to the assumption that they may be at a higher risk of experiencing severe irAEs or autoimmune disease exacerbations. Nonetheless, in real-world clinical practice, patients with underlying AI conditions are sometimes treated with ICIs, particularly when other treatment options are limited. Although numerous studies reported the outcomes of ICI use in such patients, most of them are observational and retrospective, thereby limiting the objective assessment of the risk associated with ICI use in this population [4].

In this study, we retrospectively analyzed risk factors for irAEs associated with ICIs in real-world settings, with a particular focus on adverse events related to AI. The cohort comprised patients with various types of malignancies treated with ICIs covered by health insurance, including different treatment regimens and combinations with other agents. We evaluated the risk of AI, discontinuation of ICI due to irAEs while adjusting for confounding factors, and the impact of AI on irAE-free survival. Additionally, we investigated the characteristics of irAEs observed in cases where AI was present.

## Methods

### Study design

This cohort study included patients who received ICIs at the Shiga University of Medical Science Hospital from April 1, 2017, to May 31, 2022. The inclusion criteria were patients with any type of cancer who were 18 years or older at the start of ICI treatment. Patients who received only one course of ICI due to reasons other than irAEs were excluded. In such cases, disease conditions were too unstable to continue the therapy. In cases where patients received different ICIs at different periods, each treatment record was considered an independent case. The observation period extended for at least 6 months after the last ICI dose. The data cut-off was done Jun 29, 2023. The following information was extracted from the medical records: gender, age, performance status (PS), cancer type, ICI type, type of combined drugs, best overall response, presence of pre-existing AI, type of AI, drugs used for AI at the time of ICI initiation, occurrence of irAE, type of irAE, irAE grade according to the Common Terminology Criteria for Adverse Events (CTCAE) version 5.0, date of ICI initiation, date of the last ICI administration, date of irAE occurrence, and outcome of irAE (whether the ICI was stopped or continued). The response to ICI was evaluated by attending physicians, with assessments typically conducted every 3–4 months based on individual patient conditions. Patients who discontinued ICI before the first response evaluation were classified as NE (not evaluable).

This study was reviewed and approved by the Ethics Committee of the Shiga University of Medical Science (ethical approval number: R2021-060). Informed consent was obtained through a web-based opt-out process. Under the approval, data collection from medical records was conducted by having access to the minimum information that could identify individual participants. The obtained individual records were handled by adding an anonymous research ID to each record to avoid direct recognition of individuals during the analysis. The table indicating the correspondence between research ID and medical record ID was password-locked and securely stored. The research was conducted in compliance with the Declaration of Helsinki.

### Statistical analysis

#### Covariates and definitions

The following clinical factors were used as covariates: gender, age, PS, cancer type, ICI type, type of combined drugs, best overall response, and presence or absence of pre-existing AI. Gender was coded as binary data (female = 0, male = 1). The ordinal variable PS was categorized as follows: R (reference), PS 0; C1, PS 1; C2, PS 2–4. PS 2–4 was grouped into one category due to a smaller number compared to PS 0 or 1. The nominal variable cancer type was categorized as follows: R (reference), melanoma; C1, lung cancer; C2, digestive tract cancer; C3, head and neck cancer; C4, renal cell cancer; C5, urothelial cancer; C6, liver cancer; C7, other cancers (Merkel cell cancer, uterine cancer, MSI-H tumor, malignant mesothelioma, malignant lymphoma). Melanoma was chosen as the reference category due to its highest percentage of irAE occurrence. ICI types were categorized as follows: R, anti-PD-1 and anti-CTLA4 antibodies; C1, anti-PD-1 antibody; C2, anti-PD-L1 antibody. Only two cases (both melanoma cases) were treated with anti-CTLA4 Ab alone, and this category was excluded from the analysis. Types of combined drugs were categorized as follows: R, none; C1, cytotoxic agents; C2, molecular targeted agents. Best overall responses were categorized as follows: R, complete response (CR) + partial response (PR); C1, stable disease (SD); C2, progressive disease (PD); C3, not evaluable (NE). Since the non-CR/non-PD case comprised only one, this category was excluded from the analysis. Since the number of CR cases was small (n = 25), it was combined with PR as one category. Pre-existing AI was coded as binary data (absence = 0, presence = 1). Univariate and multivariate analyses were used to calculate odds ratios (ORs) and 95% confidence intervals (CIs).

#### Study outcomes

The occurrence of irAEs was converted into binary data (no irAE occurred = 0, any irAE occurred = 1) as the dependent variable. Univariate and multivariate analyses were performed to identify factors significantly associated with the occurrence of irAEs. Potential confounding factors among the covariates were adjusted by using multivariate logistic regression analysis. Discontinuation of ICI after irAE occurrence was also considered as a dependent factor, which was converted into binary data (ICI was continued = 0, ICI was stopped = 1). The covariates were the same as mentioned above. The odds ratios from univariate and multivariate analyses as well as 95% confidence intervals were determined and presented as forest plots for the risk of irAE occurrence or the discontinuation of ICI after irAE occurrence.

The time from ICI administration to the onset of irAE or death due to any reason, whichever occurs first, was considered as an event. The irAE-free survival was compared between the AI (-) and AI (+) groups using the Kaplan-Meier method and analyzed with the log-rank test.

All statistical analyses were performed using IBM SPSS Statistics version 25.

## Results

### Patient characteristics

**Fig 1:**
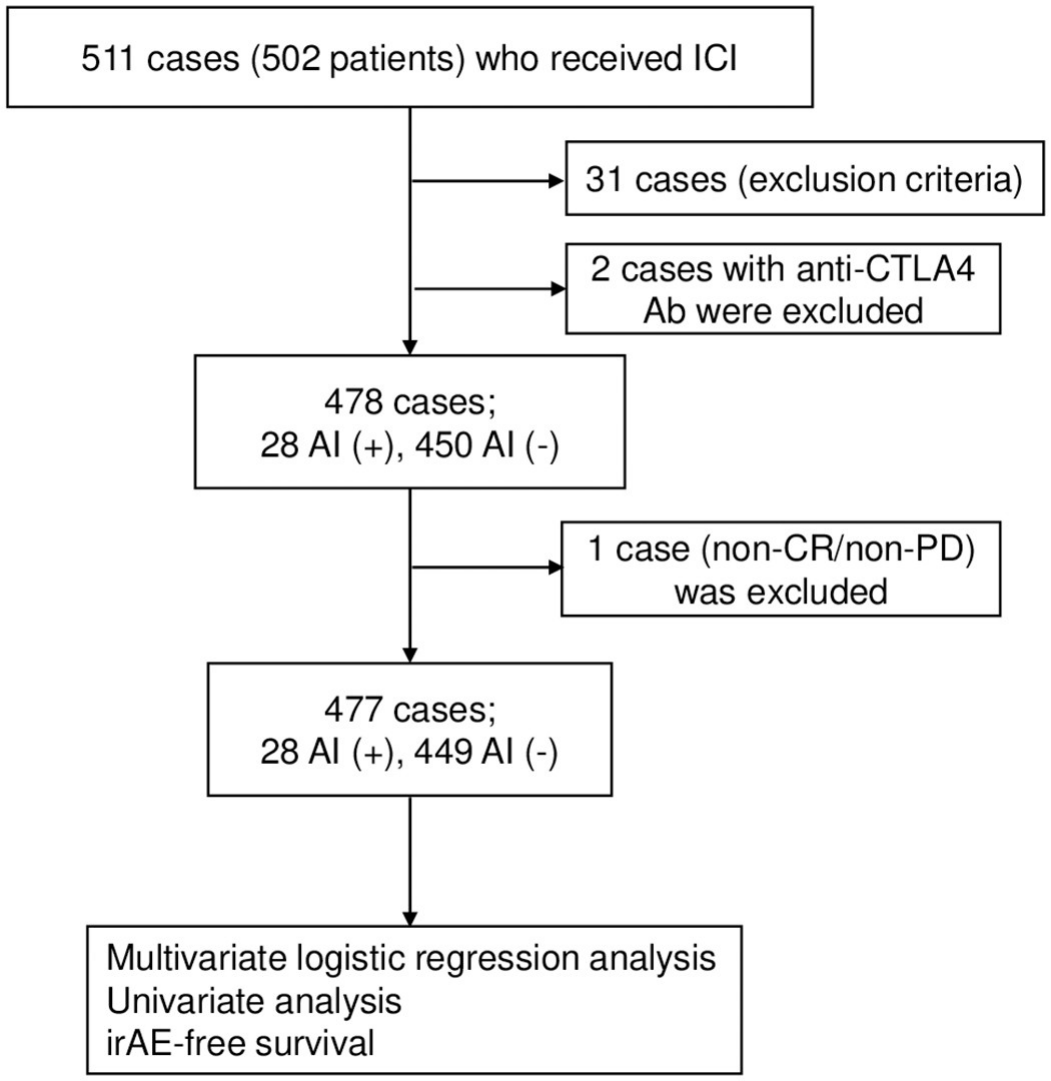
Flow chart of patient selection.

Between April 1, 2017, and May 31, 2022, a total of 511 cases (502 patients) received ICI treatment at our hospital. Among them, 31 cases met the exclusion criteria. Two cases were treated with anti-CTLA4 Ab alone, which were removed from the analysis. Therefore, a total of 478 cases were included in the analysis (one non-CR/non-PD case was excluded during the analysis). The flow chart of patient selection is shown in Fig 1. Among these, 28 cases (5.9% of all cases) had a history of AI, while 450 cases did not. Table 1 summarizes the patient characteristics. The incidence of irAE was higher in the AI (+) group (57.1%) compared to the AI (-) group (34.9%). Similarly, the incidence of ICI discontinuation due to irAE was higher in the AI (+) group (75.0%) compared to the AI (-) group (59.7%). However, the contribution of AI history to the occurrence of irAE or ICI discontinuation may be confounded by different backgrounds.

**Table 1:** Patient characteristics between AI (+) and AI (-) groups.

	AI (+), n (%)	AI (-), n (%)
**N**	28	450
**Gender (male)**	19 (67.9)	340 (75.6)
**Age (median)**	69 (47–90)	70 (34–87)
**PS**		
0	13 (46.4)	210 (46.7)
1	11 (39.3)	194 (43.1)
2	4 (14.3)	31 (6.9)
3	0 (0.0)	14 (3.1)
4	0 (0.0)	1 (0.2)
**Type of cancer**		
Lung	15 (53.6)	151 (33.6)
Digestive tract	1 (3.6)	75 (16.7)
Head & Neck	2 (7.1)	63 (14.0)
Melanoma	1 (6.3)	44 (9.8)
Renal	0 (0.0)	41 (9.1)
Urothelial	3 (10.7)	31 (6.9)
Liver	2 (7.1)	29 (6.4)
Others	4 (14.3)	16 (3.6)
**Type of ICI**		
Anti-PD-1 Ab	18 (64.3)	363 (80.7)
Anti-PD-L1 Ab	9 (32.1)	70 (15.6)
Anti-CTLA4	0 (0.0)	2 (0.4)
Anti-PD-1& Anti-CTLA4 Ab	1 (3.6)	15 (3.3)
**Combined agent**		
None	20 (71.4)	341 (75.8)
Cytotoxic agents	6 (21.4)	68 (15.1)
Molecular target agents	2 (7.1)	41 (9.1)
**Best overall response**		
CR	2 (7.1)	23 (5.1)
PR	11 (39.3)	104 (23.1)
SD	8 (28.6)	127 (28.2)
Non-CR/non-PD	0 (0.0)	1 (0.2)
PD	4 (14.3)	54 (12.0)
NE	3 (10.7)	1 (8.3)
**irAE occurrence**		
(-)	12 (42.9)	293 (65.1)
(+)	16 (57.1)	157 (34.9)
**G3≧ irAE**	6 (21.4)	66 (14.7)
**ICI stop due to irAE**	12 (75.0)*	92 (59.7)*
**Death** **	12 (42.9)	231 (51.3)

*% of irAE (+) cases,

** death observed until 2023/06/29

### Factors associated with irAE occurrence and ICI discontinuation due to irAEs

We performed multivariate logistic regression analysis to identify risk factors associated with the occurrence of irAEs, while adjusting for confounding factors. The following eight clinical factors were selected as independent variables: gender, age, PS, type of cancer, type of ICI, combined anti-neoplastic agent, best overall response, and AI history. For ordinal or nominal variables with more than two categories (PS, type of cancer, type of ICI, combined anti-neoplastic agent, best overall response), a reference category was established based on the most frequently observed irAE occurrence, except for combined anti-neoplastic agent (reference: none) and best overall response (reference: CR/PR) in each category (S1 Table): PS 0 (irAE occurrence: 43.5%), melanoma (irAE occurrence: 46.7%), anti-PD-1 and anti-CTLA4 antibodies (irAE occurrence: 44.4%). An outlier, represented by a single record with non-CR/non-PD response, was excluded from the analysis. Fourteen of the NE cases (n = 57) involved patients receiving adjuvant ICI without target lesions.

**Fig 2:**
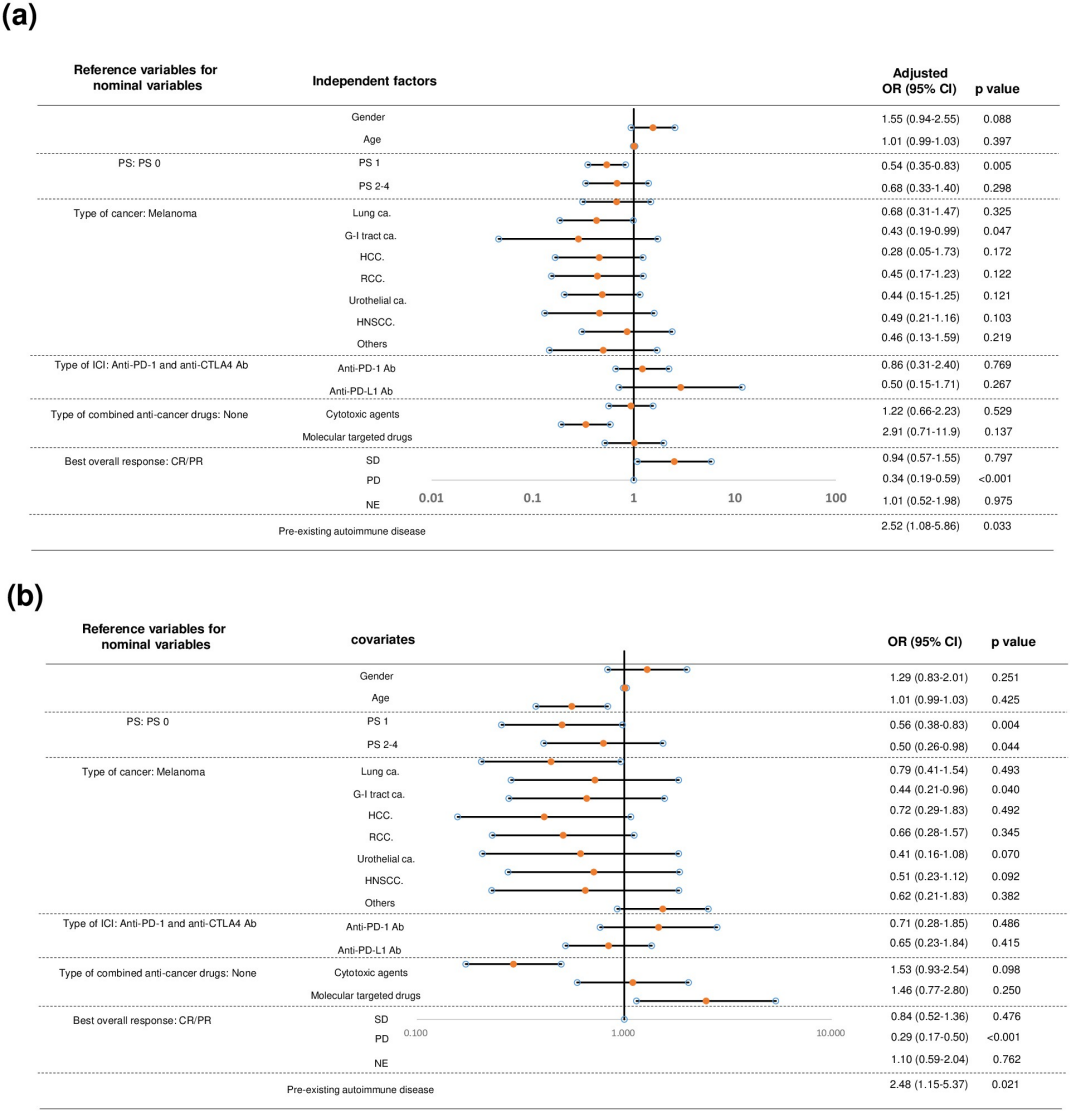
Forest plot of the association between potential risk factors and immune-related adverse events. (a) Multivariate logistic regression analysis. (b) Univariate analysis. *OR* odds ratio; *CI* confidence interval; *HCC* hepatocellular carcinoma; *RCC* renal cell carcinoma; *HNSCC* head and neck squamous cell carcinoma; *CR* complete response; *PR* partial response; *SD* stable disease; *PD* progressive disease; *NE*; not evaluable.

The factors significantly associated with the incidence of irAEs were as follows: PS 1 (compared to PS 0, adjusted OR 0.54 [95% CI: 0.35–0.83], p = 0.005), G-I tract cancer (compared to melanoma, adjusted OR 0.43 [95% CI: 0.19–0.99], p = 0.047), PD (compared to CR/PR, adjusted OR 0.34 [95% CI: 0.19–0.59], p<0.001), and pre-existing AI (adjusted OR 2.52 [95% CI: 1.08–5.86], p = 0.033). The adjusted odds ratios, except for AI history, were less than 1, indicating that PS 0, melanoma, and CR/PR were significant risk factors compared to PS 1, G-I tract cancer, and PD, respectively (Fig 2a). Subdivision of the NE category into “NE with adjuvant ICI (n = 14)” and “NE without adjuvant ICI (n = 43)” or exclusion of the latter category did not change the significance of AI (data not shown).

Since the numbers of some categories are small, such categories might be inadequately analyzed by logistic regression analysis. Therefore, we also conducted univariate analysis with the covariates. We confirmed similar results as for irAE occurrence: PS 1 (compared to PS 0, OR 0.56 [95% CI: 0.38–0.83], p = 0.004), G-I tract cancer (compared to melanoma, OR 0.44 [95% CI: 0.21–0.96], p = 0.04), PD (compared to CR/PR, OR 0.29 [95% CI: 0.17–0.50], p<0.001), and pre-existing AI (OR 2.48 [95% CI: 1.15–5.37], p = 0.021). In the univariate analysis, PS 2–4 was also found to be significant (compared to PS 0, OR 0.50 [95% CI: 0.26–0.98], p = 0.044). The significant covariates in both multivariate and univariate analyses appears to be robust (Fig 2b).

**Fig 3:**
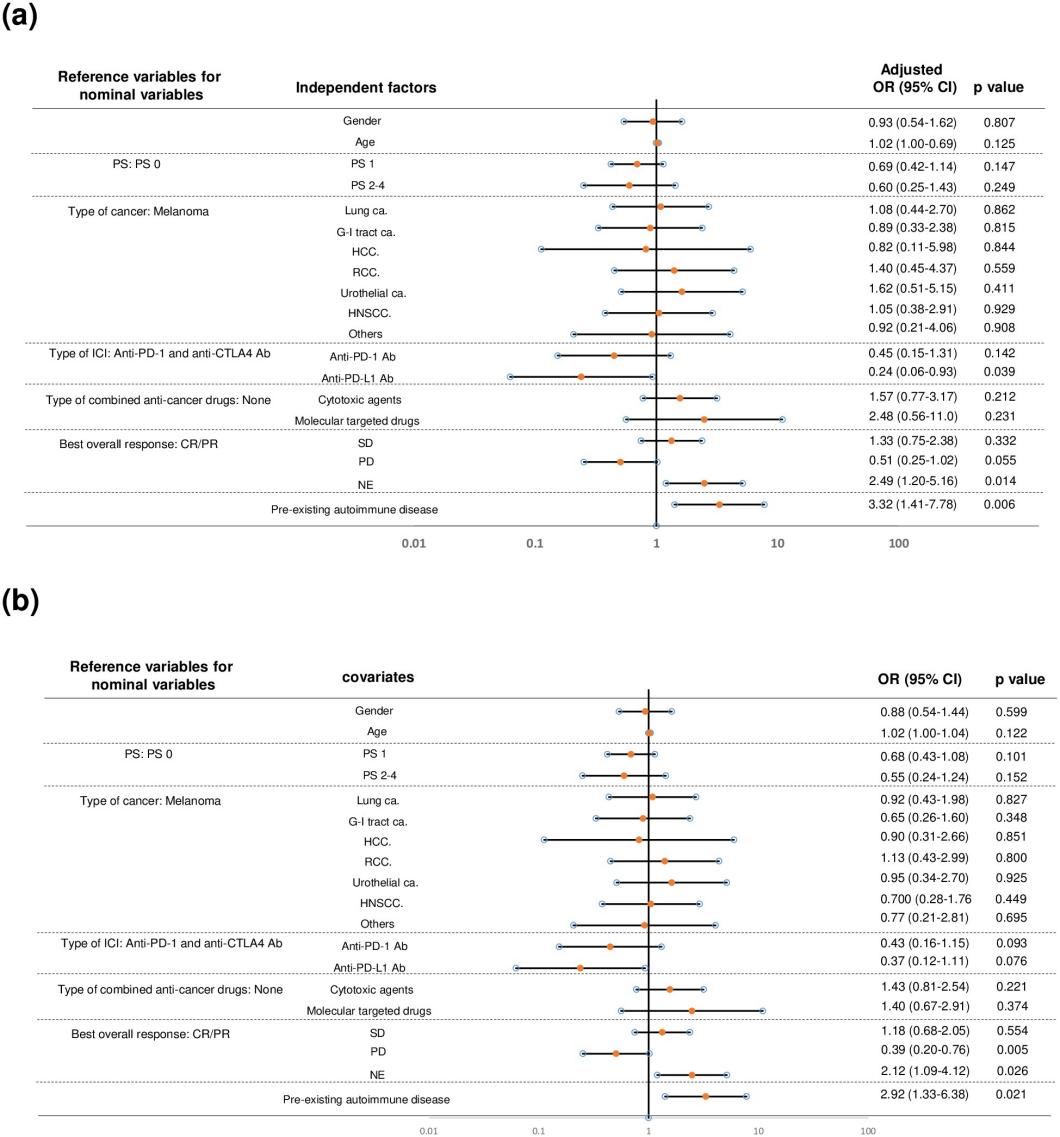
Forest plot of the association between potential risk factors and ICI discontinuation due to irAE. (a) Multivariate logistic regression analysis. (b) Univariate analysis. The abbreviations are the same with Fig 1.

Factors associated with the discontinuation of ICI due to irAEs were as follows: anti-PD-L1 Ab (compared to anti-PD1 Ab and anti-CTLA4 Ab, adjusted OR 0.24 [95% CI: 0.06–0.93], p = 0.039), NE (compared to CR/PR, adjusted OR 2.49 [95% CI: 1.20–5.16], p = 0.014), and pre-existing AI (adjusted OR 3.32 [95% CI: 1.41–7.78], p = 0.006). The adjusted odds ratio of anti-PD-L1 Ab was less than 1, indicating that anti-PD1 Ab and anti-CTLA4 Ab were significant risk factors compared to anti-PD-L1 Ab (Fig 3a). Univariate analysis also showed similar results as the multivariate analysis: NE (compared to CR/PR, OR 2.12 [95% CI: 1.09–4.12], p = 0.026), and pre-existing AI (OR 2.92 [95% CI: 1.33–6.38], p = 0.021). In the univariate analysis, PD was found to be significant (compared to CR/PR, OR 0.39 [95% CI: 0.20–0.76], p = 0.005), and PD-L1 was not significant (compared to anti-PD1/anti-CTLA4 Ab, OR 0.37 [95% CI: 0.12–1.11, p = 0.076] (Fig 3b).

### The onset of irAEs occurred earlier in patients with a history of AI compared to those without AI history

**Fig 4:**
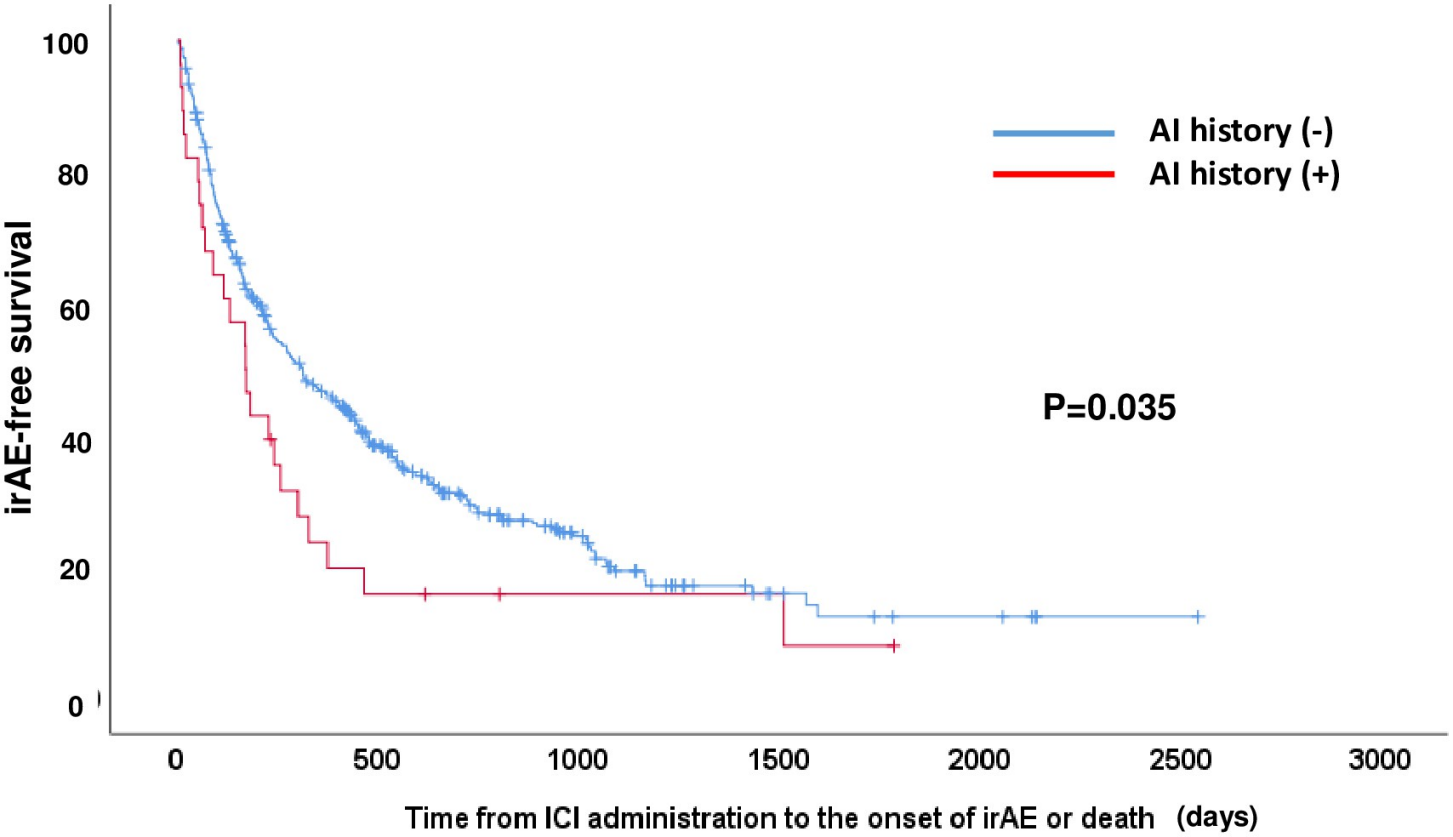
Kaplan-Meier curve of irAE-free survival between the patients with pre-existing autoimmune disease (AI) and those without it. The onset of irAE or death due to any reasons was considered as an event, whichever comes first. The median irAE-free survival time was significantly shorter in the AI (+) group compared to the AI (-) group (5.7 months [95% CI: 3.5–7.8] vs. 10.4 months [95% CI: 7.90–12.9], p = 0.035). AI (+) group; n = 28 (censored case 4), AI (-) group; n = 450 (censored case 135).

The time from ICI initiation to the onset of irAEs or death was compared between the AI (-) and AI (+) groups using the Kaplan-Meier method. The median irAE-free survival time was significantly shorter in the AI (+) group compared to the AI (-) group (5.7 months [95% CI: 3.5–7.8] vs. 10.4 months [95% CI: 7.9–12.9], p = 0.035) (Fig 4). The numbers of deaths in the AI (+) and (-) groups at the time of data cutoff were presented in Table 1. Twelve deaths (12/28; 42.9%) occurred in the AI (+) group, and 231 deaths (231/450; 51.3%) were observed in the AI (-) group. This suggests that the shorter irAE-free survival in the AI (+) group does not stem from a higher incidence of death but rather from the earlier onset of irAEs.

### Type of irAEs in all cases and their relationship to AI history

The Table 2 presents the type and CTCAE grade of irAEs observed in all cases treated with ICI, along with the number of cases with second irAE and AI history (+) for each type of irAE. Dermatologic irAEs (mostly dermatitis) (7.5%), interstitial pneumonia (7.1%), hepatitis (4.6%), and hypothyroidism (4.2%) were frequently observed. These commonly observed irAEs tended to be associated with second irAEs and AI history. However, due to their low frequencies in the total population, logistic regression analysis to determine the risk factors was not conducted for these irAEs.

**Table 2:** Type of irAEs and the grades in all cases.

Type of irAEs^1^	n (%)^2^	G1(%)^3^	G2 (%)^3^	G3 (%)^3^	G4 (%)^3^	G5 (%)^3^	2nd irAE (+)	AI history (+)
Dermatologic	36 (7.5)	12 (33.3)	18 (50.0)	6 (16.7)	0 (0)	0 (0)	10	4
Interstitial pneumonia	34 (7.1)	14 (41.2)	10 (29.4)	9 (26.5)	0 (0)	1 (2.9)	5	4
Hepatitis	22 (4.6)	1 (4.5)	1 (4.5)	15 (68.2)	5 (22.7)	0 (0)	5	3
Hypothyroidism	20 (4.2)	5 (25.0)	15 (75.0)	0 (0)	0 (0)	0 (0)	4	2
Pituitary dysfunction	9 (1.9)	0 (0)	4 (44.4)	4 (44.4)	1 (11.1)	0 (0)	2	0
Enteritis	8 (1.7)	1 (12.5)	2 (25.0)	5 (62.5)	0 (0)	0 (0)	1	0
Myositis	6 (1.3)	3 (50.0)	1 (16.7)	2 (33.3)	0 (0)	0 (0)	3	0
Hyperthyroidism	6 (1.3)	2 (33.3)	4 (66.7)	0 (0)	0 (0)	0 (0)	0	0
Nephritis	5 (1.0)	0 (0)	2 (40.0)	2 (40.0)	1 (20.0)	0 (0)	1	0
Type I diabetes	4 (0.8)	0 (0)	0 (0)	4 (100)	0 (0)	0 (0)	1	0
Encephalitis	4 (0.8)	1 (25.0)	0 (0)	3 (75.0)	0 (0)	0 (0)	0	0
Blood disorder	2 (0.4)	0 (0)	1 (50.0)	0 (0)	1 (50.0)	0 (0)	0	1
Adrenal insufficiency	2 (0.4)	0 (0)	1 (50.0)	1 (50.0)	0 (0)	0 (0)	1	0
Meningitis	2 (0.4)	0 (0)	0 (0)	2 (100)	0 (0)	0 (0)	1	1
Peripheral neuropathy	2 (0.4)	1 (50.0)	0 (0)	1 (50.0)	0 (0)	0 (0)	1	0
Arthritis	2 (0.4)	0 (0)	1 (50.0)	1 (50.0)	0 (0)	0 (0)	1	0
Interstitial cystitis	1 (0.2)	0 (0)	1 (100.0)	0 (0)	0 (0)	0 (0)	0	0
Rheumatoid arthritis	1 (0.2)	0 (0)	1 (100.0)	0 (0)	0 (0)	0 (0)	0	1
Increased amylase	1 (0.2)	0 (0)	0 (0)	0 (0)	1 (100.0)	0 (0)	0	0
Carditis	1 (0.2)	0 (0)	0 (0)	1 (100.0)	0 (0)	0 (0)	1	0
Myasthenia gravis	1 (0.2)	0 (0)	0 (0)	1 (100.0)	0 (0)	0 (0)	1	0
Cholangitis	1 (0.2)	0 (0)	0 (0)	0 (0)	1 (100.0)	0 (0)	0	0
Laryngeal edema	1 (0.2)	0 (0)	1 (100.0)	0 (0)	0 (0)	0 (0)	0	0

^1^. For the patients with second irAE, the first irAEs are indicated.

^2^. % in all ICI-treated patiens,

^3^. % in the irAE

Grade is based on Common Terminology Criteria for Adevers Events (CTCAE) Ver.5.0.

### Patient characteristics with pre-existing AIs

Among the 28 cases with AI history, irAE occurred in 16 cases (57.1%). Logistic regression analysis could not determine significant risk factors for irAEs in this population, possibly due to the small sample size (data not shown). A comparison of patient characteristics between the AI (+) and (-) groups is presented in Table 3. The frequencies of ongoing treatment against AI at the time of ICI initiation were similar between the two groups (AI history (+) 50.0% vs. AI history (-) 58.3%), suggesting that the activity of AI did not appear to correlate with irAE occurrence. The overall response rate (ORR: CR/PR) and disease control rate (DCR: CR/PR/SD) tended to be higher in irAE (+) cases compared to irAE (-) cases: ORR 50% vs. 41.7%, DCR 81.3% vs. 66.7%, respectively. However, in such a small sample size, the correlation is not definitive. The types of AIs were diverse, and no specific AI could be concluded to be related to the risk of irAEs.

**Table 3:** Comparison of the patient characteristics between irAE (+) and (-) groups among the 28 patients accompanied with autoimmune diseases (AI).

	irAE (+), n (%)	irAE (-), n (%)
**N**	16 (57.1)	12 (42.9)
**Gender (male)**	12 (75.0)	7 (58.3)
**Age (median)**	70.5 (47–90)	66 (51–90)
**PS**		
0	7 (43.8)	6 (50.0)
1	6 (37.5)	5 (41.7)
2	3 (18.8)	1 (8.3)
3	0 (0.0)	0 (0.0)
4	0 (0.0)	0 (0.0)
**Type of cancer**		
Lung	10 (62.5)	5 (41.7)
Digestive tract	0 (0.0)	1 (8.3)
Head & Neck	0 (0.0)	2 (16.7)
Melanoma	1 (6.3)	0 (0.0)
Urothelial	1 (6.3)	2 (16.7)
Liver	2 (12.5)	0 (0.0)
Merkel cell ca.	2 (12.5)	0 (0.0)
Malignant mesothelioma	0 (0.0)	1 (8.3)
MSI-H tumor	0 (0.0)	1 (8.3)
**Type of ICI**		
Anti-PD-1 Ab	9 (56.3)	9 (75.0)
Anti-PD-L1 Ab	7 (43.8)	2 (16.7)
Anti-PD-1& Anti-CTLA4 Ab	0 (0.0)	1 (8.3)
**Combined agent**		
None	10 (62.5)	10 (83.3)
Cytotoxic agents	4 (25.0)	2 (16.7)
Molecular target agents	2 (12.5)	0 (0.0)
**Best overall response**		
CR	1 (6.3)	1 (8.3)
PR	7 (43.8)	4 (33.3)
SD	5 (31.3)	3 (25.0)
PD	1 (6.3)	3 (25.0)
NE	2 (12.5)	1 (8.3)
**Type of AI**		
RA	5 (25.0)	6 (50.0)
Hashimoto’s thyroiditis	4 (20.0)	0 (0.0)
Basedow’s disease	1 (5.0)	2 (16.7)
SLE*	2 (10.0)	0 (0.0)
SjS	0 (0.0)	1 (8.3)
ITP	2 (10.0)	1 (8.3)
Pemphigoid	1 (5.0)	0 (0.0)
Psoriasis vulgaris	1 (5.0)	0 (0.0)
PBC	1 (5.0)	0 (0.0)
Palmoplantar pustulosis	1 (5.0)	0 (0.0)
UC	2 (10.0)	1 (8.3)
Crohn’s disease	0 (0.0)	1 (8.3)
**Type of drug for AI at the time of ICI start**		
Steroid	4 (25.0)	2 (16.7)
Methotrexate	1 (6.3)	1 (8.3)
Others	3 (18.8)	4 (33.3)
None	8 (50.0)	5 (41.7)

* accompanied with scleroderma

PBC; primary biliary cirrhosis, UC; ulcerative colitis, SjS; Sjogrens’s syndrome, ITP; idiopathic thrombocytopenic purpula

**Fig 5:**
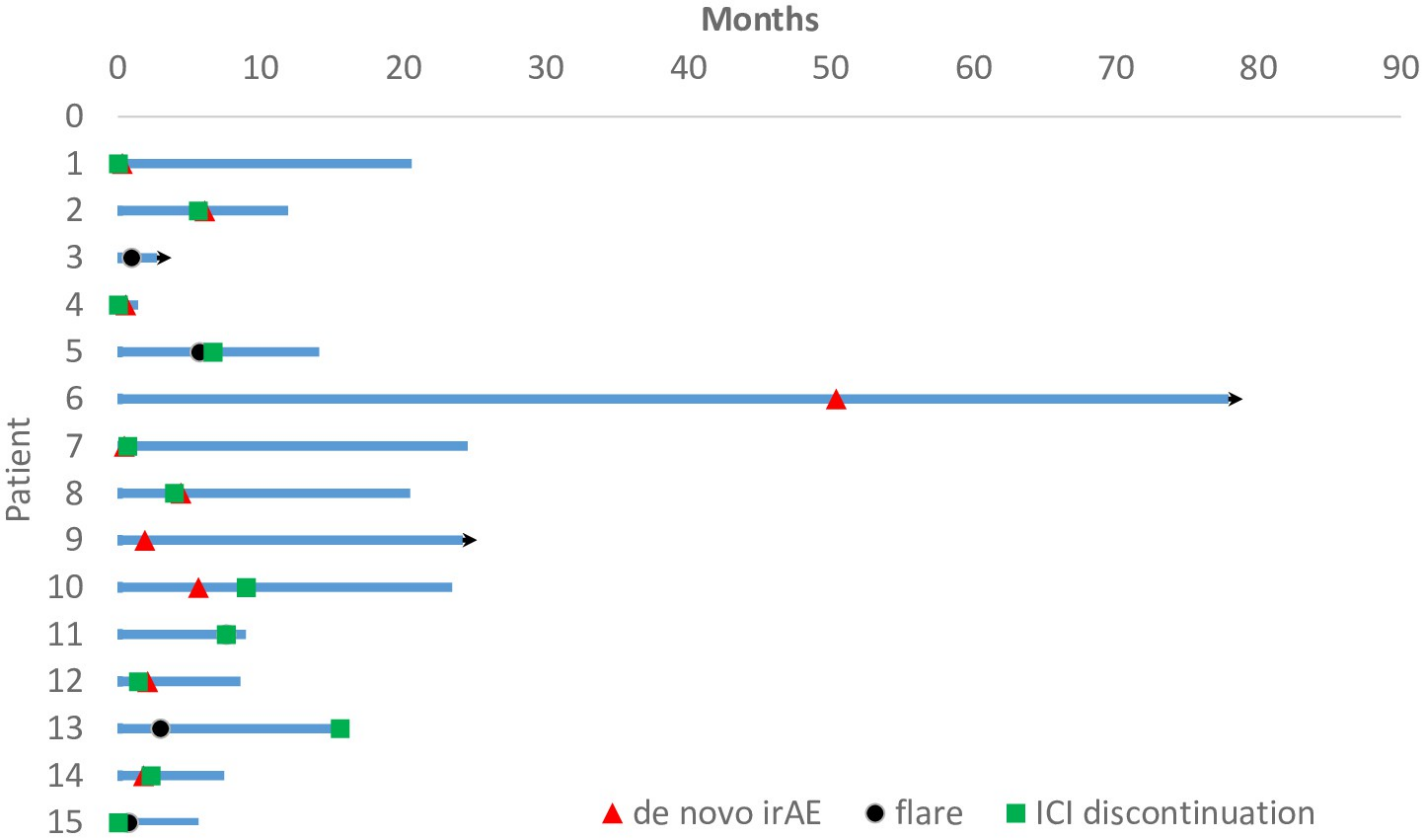
Swimmer’s plot of irAE (+) cases in AI (+) patients. Red rectangle; onset of *de novo* irAE, black circle; onset of AI flare, green square; the time of ICI discontinuation due to irAE, arrow head; ICI continuation at the time of data cut-off. One re-challenged case (no. 13) was omitted because continuation of AI flare at the initiation of the second ICI challenge.

Further characterization of irAEs in the 16 AI (+) cases is presented in Table 4. The table summarizes the type of irAEs, the number of cases for each irAE, CTCAE grades, flare of pre-existing AI, and ICI discontinuation due to irAEs. Flare cases were found in 6 out of 16 cases (37.5%), and *de novo* irAEs were more frequent than flares, suggesting that pre-existing AI is a non-specific risk factor for irAEs. Fig 5 shows the swimmer’ plot of irAE(+) cases in AI (+) patients, which indicates the timing of flares or de novo irAE during the course. It should be noted that some irAEs (interstitial pneumonia, hypothyroidism, rheumatoid arthritis) led to the discontinuation of ICI with grade 2, indicating a tendency to stop ICI early in cases with AI history.

**Table 4:** Type and grade of irAEs in AI (+) cases.

Type of irAEs^1^	n	G1	G2	G3	G4	Flare of AI	ICI stop due to irAE
Dermatitis^2^	4	0	3	1	0	1	2
Interstitial pneumonia	5	1	4	0	0	0	4
Hepatitis^3^	3	0	0	2	1	1	3
Hypothyroiidism	4	0	4	0	0	2	1
Meningitis	1	0	0	1	0	0	1
RA	1	0	1	0	0	1	1
ITP	1	0	0	0	1	1	0
Pituitary dysfunction	1	0	2	0	0	0	0

^1^. Four cases had multiple irAEs; hepatitis + IP (1), dermatitis +thyroiditis (2), dermatitis + pituitary dysfunction (1).

^2^. Exacerbation of pre-existed psoriasis vulgaris (1).

^3^. Exacerbation of pre-existed rpimary biliary cirrhosis (1).

## Discussion

Here, we retrospectively reviewed the incidence, severity, and types of irAEs in cancer patients receiving ICI at our institution over a relatively short period of 5 years. This period starts from the initial approval of ICIs and includes subsequent years during which the application of ICIs has evolved, such as approval in the first-line or adjuvant setting after radiation or surgery, as well as their combined use with cytotoxic or molecular targeted agents. The mode of ICI use in clinical practice may continue to change, which could impact the occurrence of irAEs. However, the mechanism of irAEs is based on the reactivation of autoreactive T cells through the suppression of peripheral tolerance by ICIs, which appears to be universal regardless of changes in the mode of ICI application. Therefore, our analyses can provide valuable insights for evidence-based medicine, despite their limitations, by presenting the real-world status of irAEs in diverse conditions, including almost all types of cancer, various clinical backgrounds, different treatment lines, and combinations with other therapeutic modalities.

We focused on the significance of pre-existing AIs as a risk factor for irAEs. Although many studies have been published assessing the safety of ICIs in patients with AIs, most of them were observational retrospective series, and only a few studies have adjusted for confounding factors [4–6]. In our study, we confirmed that pre-existing AI was an independent risk factor for irAEs after adjusting for confounding factors, consistent with previous reports. Interestingly, the OR for AI in relation to irAE occurrence was consistently around 2–2.5 [7–9], regardless of selection bias. Major limitation of our analysis might be a small number of AI-positive cases (28/478; 5.9%). However, our analysis revealed statistical significance of AI positivity as a risk factor. This finding ensures the statistical power of our analysis and also implies an impact of AI on irAE and its outcome.

In our study, we observed flare-ups of pre-existing AI in 6 cases (37.5% in 16 AI (+) irAE (+) and 21.4% in all 28 AI (+) cases), which was similar to most previous reports [4]. Although it is difficult to interpret the risk of immune-related adverse events (irAEs) for rare types of autoimmune diseases, particularly dermatologic conditions, such as pemphigoid, psoriasis vulgaris, and palmoplantar pustulosis (each with a sample size of 1), this indicates that not only AI flare-ups but also *de novo* irAEs should be carefully monitored in patients with pre-existing AI. In our series, we did not find any correlations between the type of AI and the incidence of irAEs, although literature suggests certain correlations in specific AIs and flares, especially in rheumatic and dermatological diseases, although recruitment bias and reporting bias may exist [10, 11]. Pre-existing AI was also identified as an independent risk factor for ICI discontinuation (Fig 2), even though 7 out of 12 (58.3%) cases of ICI discontinuation had grade 2 or lower irAEs (Table 4). Grade 3/4 irAEs were found in 6 out of 20 (30.0%) AI (+) irAE (+) cases, and no grade 5 irAEs were observed, suggesting that the majority of irAEs found in AI (+) cases were mild and manageable, as previously noted [4]. The median irAE-free survival was shorter in the AI (+) group compared to the AI (-) group (5.7 vs. 10.4 months, p = 0.035) due to the earlier onset of irAE in our series, which is consistent with a previous report (5.4 vs. 13 months) [12]. Our analysis revealed several characteristics of irAEs in patients with pre-existing AI.

The significance of the use of immunosuppressants against AI as a risk factor for irAE at the initiation of ICI treatment is still a subject of debate. Our data shows a trend towards fewer patients receiving baseline immunosuppression in cases with irAEs compared to those without irAEs (50% (8/16) vs. 58.3% (7/12)) (Table 4). A similar trend was reported in a systematic review (67% vs. 74%) [13]. However, a study on non-small cell lung cancer (NSCLC) showed a higher rate of pre-existing AI flare-ups in patients with baseline immunosuppression (36%) compared to those without it (20%) [14]. A similar result was reported in advanced melanoma and pre-existing AI: baseline immunosuppression (+) 39% vs. (-) 26% [15]. The difference in the type of AI and its activity may explain these conflicting results.

The significance of PS as a risk factor for irAEs is conflicting. PS≥2 (vs. 0–1) was found to be a significant risk factor for pneumonitis (OR 3.43 [1.01–11.71], p = 0.048) [16]. However, in a larger cohort, the OR of PS≥2 (vs. 0–1) for pneumonitis, severe irAEs, and all irAEs were 0.946 [0.441–2.145] (p = 0.946), 4.724 [1.215–14.925] (p = 0.024), and 0.598 [0.449–0.797] (p = 0.0001), respectively [5]. In our study, after adjusting for confounding factors, we observed that PS 0 (vs. 1) was a significant risk factor for irAEs. PS 0 was not confounded by the best overall response. However, we cannot exclude the possibility that unknown confounding factors might have affected our results.

Regarding treatment response, it has been reported that a better treatment response is associated with an increased risk of irAEs (OR 3.55 [2.49–5.069]) [17–20]. We also found that CR/PR (vs. PD) was a significant risk factor for irAEs, indicating that the incidence of irAEs is correlated with a better prognosis [21]. We observed a high incidence of irAEs (47.4%) in cases classified as NE (n = 57), which is difficult to interpret. Some NE cases (n = 14) lack target lesions due to the adjuvant setting. In the residual 43 NE cases, response assessment was not conducted due to loss of follow-up, assessment executed at inadequate time points, or early termination of ICI before response assessment due to worsening of general conditions. Although we identified NE as an independent risk factor for ICI discontinuation, the relationship between NE and ICI discontinuation requires further investigation.

As for type of ICI, our analysis could not show ICI combination (anti-PD1 and anti-CTLA4 Ab) as a risk factor for irAE occurrence. The safety of ICI combination therapy (two ICI agents) versus monotherapy was retrospectively compared in patients with preexisting AI. The incidence of irAEs was higher for the combination therapy than for monotherapy. However, the incidence of high-grade irAEs or AI flares was not different between the two groups, and ICI combination therapy showed a tolerable toxicity profile [22]. In our analysis, ICI combination therapy was used in one case with preexisting AI and in 15 cases without it. The lack of statistical power in our series might explain the differing results.

In summary, our study provides several clinical pieces of evidence regarding irAEs, with a particular focus on pre-existing AI. Despite the limitations of our analyses, such as being retrospective and possible selection bias due to single-institution data, our findings reflect the real-world scenario during the early stages of ICI implementation, thus contributing to evidence-based medicine. The presence of AI is an independent risk factor for irAE occurrence, ICI discontinuation, and leads to an earlier onset of irAEs. Since the factors contributing to irAE development in cases with pre-existing AI remain unknown, further investigation, including the exploration of new biomarkers, is warranted. AI flare-ups are relatively infrequent, and the majority of irAEs are mild and manageable in severity. Although complicated AIs increase the risk of irAEs, they are not necessarily contraindicated. In situations where therapeutic options are limited, ICI might be considered for patients with complicated AIs after carefully evaluating the risk-benefit ratio, and with close monitoring of irAEs. Physicians should carefully consider the indications of ICI treatment in cases with AI, based on the available evidence.

## Supporting information

S1 Table Frequency of irAE according to the categories. (PDF)
