# Supplementary material for: Pre-existing autoimmune disease as a risk factor for immune-related adverse events in cancer patients receiving immune checkpoint inhibitors
Source: PLoS One. 2024 Jul 16;19(7):e0306995. doi: 10.1371/journal.pone.0306995 (PMC11251620; doi:10.1371/journal.pone.0306995)
Supplement: S1 Table — (PDF) [file pone.0306995.s001.pdf]

	irAE (+), n	irAE (-), n	% irAE
**PS**			
0	97	126	43.5
1	62	143	30.2
2-4	14	36	28.0
**Type of cancer**			
Lung	68	98	41.0
Digestive tract	21	55	27.6
Head & Neck	20	45	30.8
Melanoma	21	24	46.7
Renal	15	26	36.8
Urothelial	9	25	26.5
Liver	12	19	38.7
Others	7	13	35.0
**Type of ICI**			
Anti-PD1	138	243	36.2
Anti-PD-L1	27	41	44.6
Anti-PD1&Anti-CTLA4	8	10	44.4
**Combined anti-neoplastic agent**			
None	122	239	33.8
Cytotoxic agents	33	41	44.6
Molecular target agents	18	25	41.9
**Best overall response**			
CR/PR	63	77	45.0
SD	55	80	40.7
Non-CR/Non-PD	0	1	0.0
PD	28	117	19.3
NE	27	30	47.4

Supplemental Table 1. Frequency of irAE according to the categories
